# Photoperiod Affects Leptin Action on the Choroid Plexus in Ewes Challenged with Lipopolysaccharide—Study on the mRNA Level

**DOI:** 10.3390/ijms21207647

**Published:** 2020-10-15

**Authors:** Aleksandra Szczepkowska, Marta Kowalewska, Agata Krawczyńska, Andrzej P. Herman, Janina Skipor

**Affiliations:** 1 Institute of Animal Reproduction and Food Research, Polish Academy of Sciences, 10-748 Olsztyn, Poland; a.szczepkowska@pan.olsztyn.pl (A.S.); marta.kowalewska89@gmail.com (M.K.); 2 The Kielanowski Institute of Animal Physiology and Nutrition, Polish Academy of Sciences, 05-110 Jabłonna, Poland; a.krawczynska@ifzz.pl (A.K.); a.herman@ifzz.pl (A.P.H.)

**Keywords:** choroid plexus, leptin, LEPRa, LEPRb, SOCS3, photoperiod, LPS, TLR4, proinflammatory cytokines, relative gene expression

## Abstract

The ovine choroid plexus (ChP) expresses the long isoform of the leptin receptor, which makes this structure a potential target for leptin action. In sheep, leptin concentration in plasma is higher during long days (LD) than short days (SD). This study evaluates the influence a of photoperiod on leptin impact on the gene expression of Toll-like receptor 4 (*TLR4*), proinflammatory cytokines (*IL1B*, *IL6*), their receptors (*IL1R1*, *IL1R2*, *ILRN*, *IL6R*, *IL6ST*) and inflammasome components necessary for pro-IL-1β activation (*NLRP3*, *PYCARD*, *CASP1*), chemokine (*CCL2*), leptin receptor isoforms (*LEPRa*, *LEPRb*) and a suppressor of cytokine signalling (*SOCS3*) in the ChP of ewes treated or not with lipopolysaccharide (LPS). Studies were conducted on adult female sheep divided into four groups (*n* = 6 in each): control, leptin (20 μg/kg), LPS (400 ng/kg), and LPS and leptin injected under SD and LD photoperiods. The leptin alone did not affect the gene expression but in co-treatment with LPS increased (*p* < 0.05) IL1B but only during SD, and *SOCS3*, *IL1R2*, *IL1RN*, *IL6ST* and *CCL2* only during LD, and decreased (*p* < 0.05) the *IL1R1* expression only during SD photoperiod. This indicates that the immunomodulatory action of leptin on the ChP is manifested only under the LPS challenge and is photoperiodically dependent.

## 1. Introduction

The choroid plexus (ChP), suspended inside the cerebral ventricles, forms an interface between blood and the cerebrospinal fluid (CSF). The ChP consists of a continuous layer of tightly connected, secretory epithelial cells overlying an extracellular stroma surrounding the vascular central core with fenestrated epithelium. Different populations of immune cells are associated with the ChP; dendritic cells and macrophages in the stroma and the Kolmer’s epiplexus cells at the apical side of the epithelial layer [1]. Being the main source of the CSF [2], the ChP is the place of the blood–CSF barrier (BCSFB) location [3,4], the source of diverse molecules, including miRNA [5,6] as well as the gateway for immune cell and pathogen entry into the brain [7,8,9]. The ChP plays an active role in the central nervous system (CNS) development [10] and function in health and disease [11,12]. The ChP and CSF system also plays an important role in the neuroendocrine regulatory loops involved in the control of seasonal functions in animals in which circannual changes in day length (photoperiod) induce seasonal physiological adaptation to the environmental living condition [13].

In sheep, living at higher latitudes, the functioning of the ChP is modulated by photoperiod. The levels of tight junction proteins in the ovine ChP are higher under short days (SD) than long days (LD) [14], which indicates differences in the BCSFB permeability underlying the differential access of peripheral hormones to the CSF. Indeed, in sheep, the higher passage of steroids [15,16,17] from the circulating blood to the CSF was observed under LD than SD. This phenomenon may also be explained by the dilution or concentration of molecules in the CSF, since the turnover rate of the CSF in sheep is higher under SD [18]. The fenestrated capillaries of the ChP are regulated by the vascular endothelial growth factor (VEGF), therefore the higher expression of VEGF receptor 2 observed in the ovine ChP during SD [19] allows for the photoperiodic plasticity of ChP capillaries. In sheep, the photoperiod also modulates the CSF proteome composition in physiological conditions [20] as well as the ChP response to lipopolysaccharide (LPS)-induced acute systemic inflammation [21].

In domestic animals, leptin, a cytokine-like polypeptide hormone produced and secreted mainly by adipocytes, links nutritional status and the innate and adaptive immune system [22]. As a hormone, leptin regulates food intake and basal metabolism, and as a cytokine, leptin affects the secretion of proinflammatory cytokines [23]. In sheep, plasma leptin concentration is sensitive to photoperiod-driven changes in food intake and adiposity and is higher during LD than SD [24]. Leptin concentration in the CSF is higher during LD and correlates with the plasma leptin level [25]. Leptin acts through a membrane-spanning leptin receptor (LEPR) that belongs to the class I cytokine receptor family [26] and exists in a few isoforms (LEPRa–f) [27]. All isoforms, except LEPRe, have the same extracellular and transmembrane domains and possess a motif binding Janus tyrosine kinase 2 (JAK2), while only LEPRb has a full-length intracellular domain with motifs necessary to activate the JAK2/signal transducer and activator of transcription (STAT) pathway [28]. The activation of the JAK2/STAT3 pathway rapidly induces the expression of the suppressor of cytokine signalling 3 (SOCS3), which inhibits receptor phosphorylation and further activation of signalling cascades [29]. LEPRa is thought to be involved in leptin transport from the periphery to the brain [30,31] and is abundant in the ChP and brain microvessels [32]. In turn, LEPRb is mainly expressed in the hypothalamus and is also present in the ChP [33,34] and all types of immune cells involved in the innate and adaptive immunity [35,36]. This makes the ChP a potential target for leptin. Numerous studies demonstrated the proinflammatory action of leptin manifested by the stimulation of proinflammatory cytokine synthesis [37,38] and the regulation of Toll-like receptor (TLR) expression in immune cells [39]. Acting at the level of brain barriers, leptin also regulates the recruitment of leukocytes to the brain during LPS-induced inflammation [40].

Considering this, we hypothesised that in sheep, leptin induces the expression of genes connected with innate immunity in the ChP under basal and LPS-challenged conditions, and that this action is modulated by photoperiod. To verify this hypothesis, we evaluated the effect of exogenous leptin on the transcription of Toll-like receptor 4 (*TLR4*), proinflammatory cytokines (interleukin-1β (*IL1B*) and interleukin-6 (*IL6*)) and their receptors (IL-1β type I (*IL1R1*) and type II (*IL1R2*) and IL-1β receptor antagonist (*IL1RN*), IL-6 receptor (*IL6R*) and IL-6 signal transducer (*IL6ST*)), C–C motif chemokine ligand 2 (*CCL2*) as well as components of inflammasome necessary for proIL-1β activation [41], which includes NLR family pyrin domain containing 3 (*NLRP3*), PYD and CARD domain-containing (*PYCARD*) and caspase 1 (*CASP1*) in the ChP of ewes treated with saline or LPS during SD and LD photoperiod. To check the effect of photoperiod and leptin on the leptin receptor and its signalling we evaluated the mRNA expression of *LEPRa* and *LEPRb* as well as *SOCS3* as a marker of JAK2/STAT3 pathway activation [42].

## 2. Results

### 2.1. The Effect of Leptin on the Body Temperature and Hormones Concentrations

**Figure 1:**
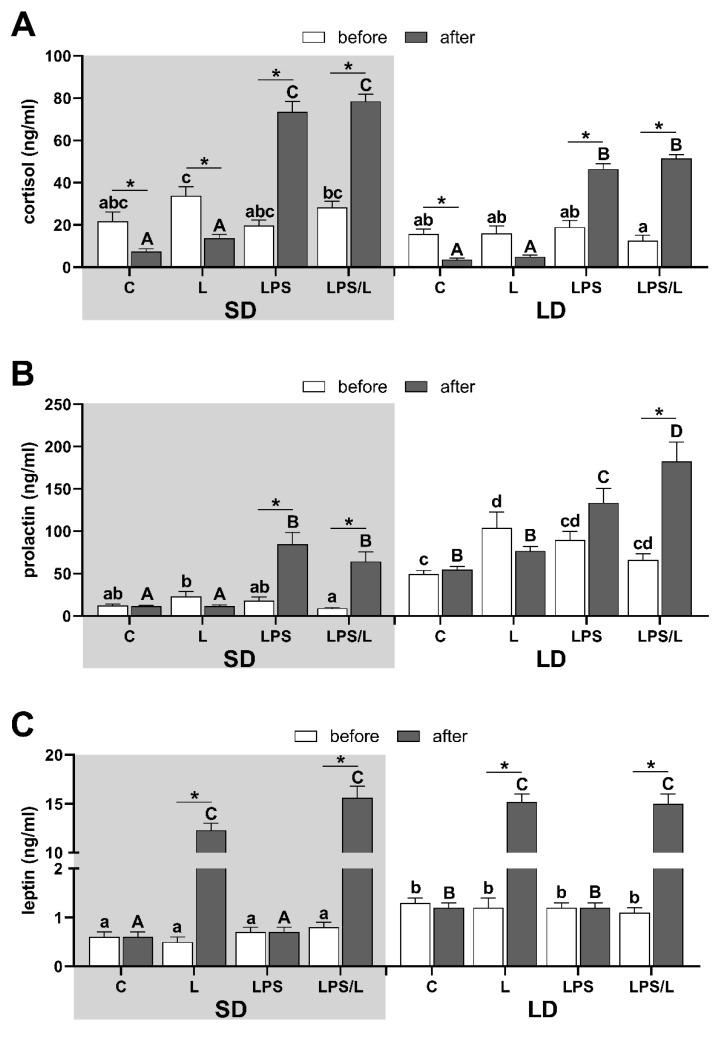
The overall mean (±SEM) concentration of cortisol (**A**), prolactin (**B**) and leptin (**C**) in blood plasma during the short days (SD, grey background) and the long days (LD, white background) in control (C), leptin-injected (L), lipopolysaccharide-injected (LPS) and LPS- and leptin-injected (LPS/L) groups, collected before (white bars) and after LPS/leptin/saline treatments (dark bars). Significant differences at *p* < 0.05, were indicated by: *—for treatment effect within the groups (repeated measures ANOVA—Sidak’s post hoc test) and by different letters for the photoperiod and treatments effects calculated separately for data before treatments (abcd—lowercase letters) and after treatments (ABCD—uppercase letters) by LPS/leptin (two-way ANOVA-Tukey’s post hoc test).

In both photoperiods, the body temperature increased following LPS administration in LPS and LPS/L groups (Supplementary Figure S1), which indicates the proper reaction of sheep to LPS treatment. This was further confirmed by the increase (*p* < 0.05) in plasma cortisol concentration in LPS-treated sheep which was higher (*p* < 0.05) during the SD than LD, and for which we did not observe any impact of leptin (Figure 1A).

Lower (*p* < 0.05) plasma prolactin concentration during SD than LD (Figure 1B) indicates a proper animal response to SD and LD conditions [43]. The leptin treatment did not affect prolactin concentration in both photoperiods. Prolactin concentration increased (*p* < 0.05) following LPS administration in LPS and LPS- and leptin-injected (LPS/L) groups during SD and LPS/L group during LD (Figure 1B). Leptin concentration in blood plasma was significantly (*p* < 0.05) higher in LD than SD in all ewes before treatment and leptin administration increased its level (*p* < 0.05) to the similar mean level in all leptin-treated groups (Figure 1C).

### 2.2. The Effect of Leptin on TLR4 Gene Expression

In the ovine ChP, the leptin treatment had no effect on TLR4 expression in both photoperiods. The TLR4 expression was upregulated following LPS administration in both photoperiods and was significantly higher under SD than LD conditions (Supplementary Figure S2).

### 2.3. The Effect of Leptin on LEPRa, LEPRb, and SOCS3 Gene Expression

**Figure 2:**
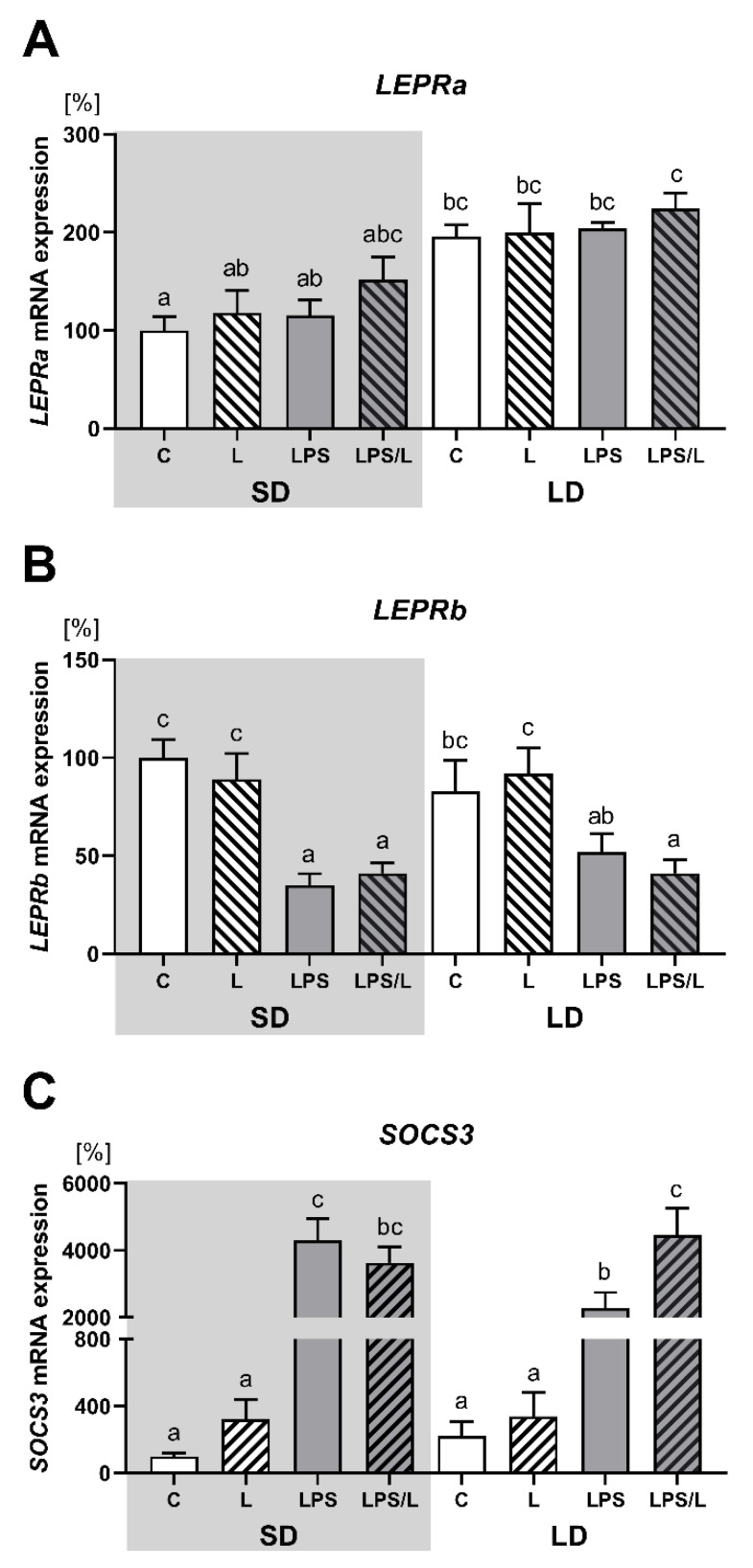
Mean (±SEM) mRNA expression of *LEPRa* (**A**), *LEPRb* (**B**) and *SOCS3* (**C**) in the ovine choroid plexus of saline (C, control—white bars), saline–leptin (L, white hatched bars), LPS (grey bars) and LPS–leptin (LPS/L, grey hatched bars) treated ewes during the short-day (SD, dark background) and the long-day (LD, light background) photoperiods. Different lowercase letters indicate significant differences between all groups at *p* < 0.05 (two-way ANOVA—Tukey’s post hoc test). The results are presented as percentage values (%), where the SD C group is considered as 100%.

The leptin injection alone and after prior LPS treatment had no effect on the expression of both LEPRa and LEPRb in the ovine ChP (Figure 2A,B, respectively). In the saline-treated group, the expression of LEPRa was significantly (*p* < 0.05) higher during LD than SD (Figure 1A). There was no effect of leptin alone, as was the case LPS and LPS in co-treatment with leptin on LEPRa expression. In turn, the expression of LEPRb was significantly (*p* < 0.05) lower in the LPS and LPS/L groups than in the saline-treated groups (C and L groups) during SD and in the LPS/L group during LD (Figure 2B).

As indicated in Figure 2C, the expression of SOCS3 was not influenced by leptin alone but was significantly (*p* < 0.05) upregulated by LPS and LPS in co-treatment with leptin in both photoperiods. However, the synergistic effect of leptin and LPS was revealed only during LD. Moreover, the increase in SOCS3 expression after LPS treatment was significantly higher during SD than LD.

### 2.4. The Effect of Leptin on IL1B, IL1R1, IL1R2 and IL1RN Gene Expression

**Figure 3:**
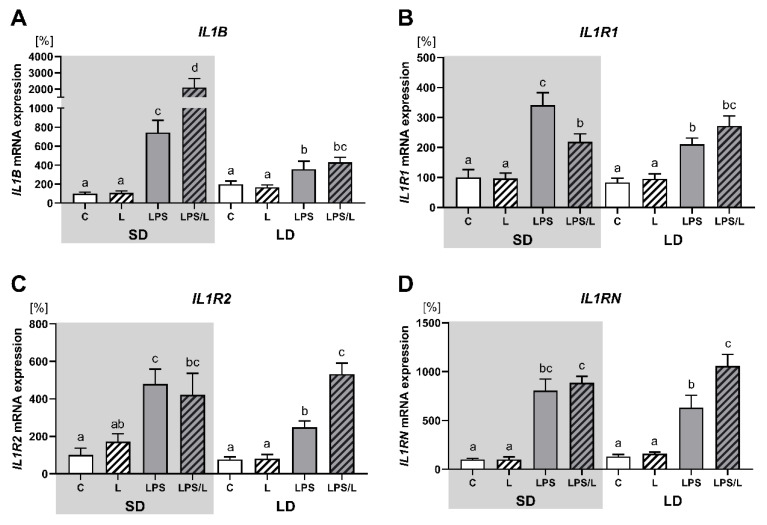
Mean (±SEM) relative *IL1B* (**A**), *IL1R1* (**B**), *IL1R2* (**C**) and *IL1RN* (**D**) mRNA expression in the ovine choroid plexus of saline (C, control—white bars), saline–leptin (L, white hatched bars), LPS (grey bars) and LPS–leptin (LPS/L, grey hatched bars) treated ewes, during the short-day (SD, dark background) and the long-day (LD, light background) photoperiod. Different lowercase letters indicate significant differences between all groups at *p* < 0.05 (two-way ANOVA—Tukey’s post hoc test). The results are presented as percentage values (%), where the SD C group is considered as 100%.

As indicated in Figure 3, leptin alone had no effect on the expression of IL1B and its receptors (IL1R1, IL1R2 and IL1RN). The expression of IL1B, IL1R1, IL1R2 and IL1RN was significantly (*p* < 0.05) upregulated by LPS and LPS in co-treatment with leptin in both photoperiods. However, the synergistic effect of leptin and LPS was revealed for IL1B only during SD (Figure 3A), and for IL1R2 and IL1RN only during LD (Figure 3C,D, respectively). In turn, LPS-induced IL1R1 mRNA expression was significantly (*p* < 0.05) attenuated by leptin during SD (Figure 3B). Moreover, the increase in the IL1B, ILR1 and ILR2 expression after LPS treatment was significantly higher during SD than LD.

### 2.5. The Effect of Leptin on NLRP3, PYCARD and CASP1 Gene Expression

**Figure 4:**
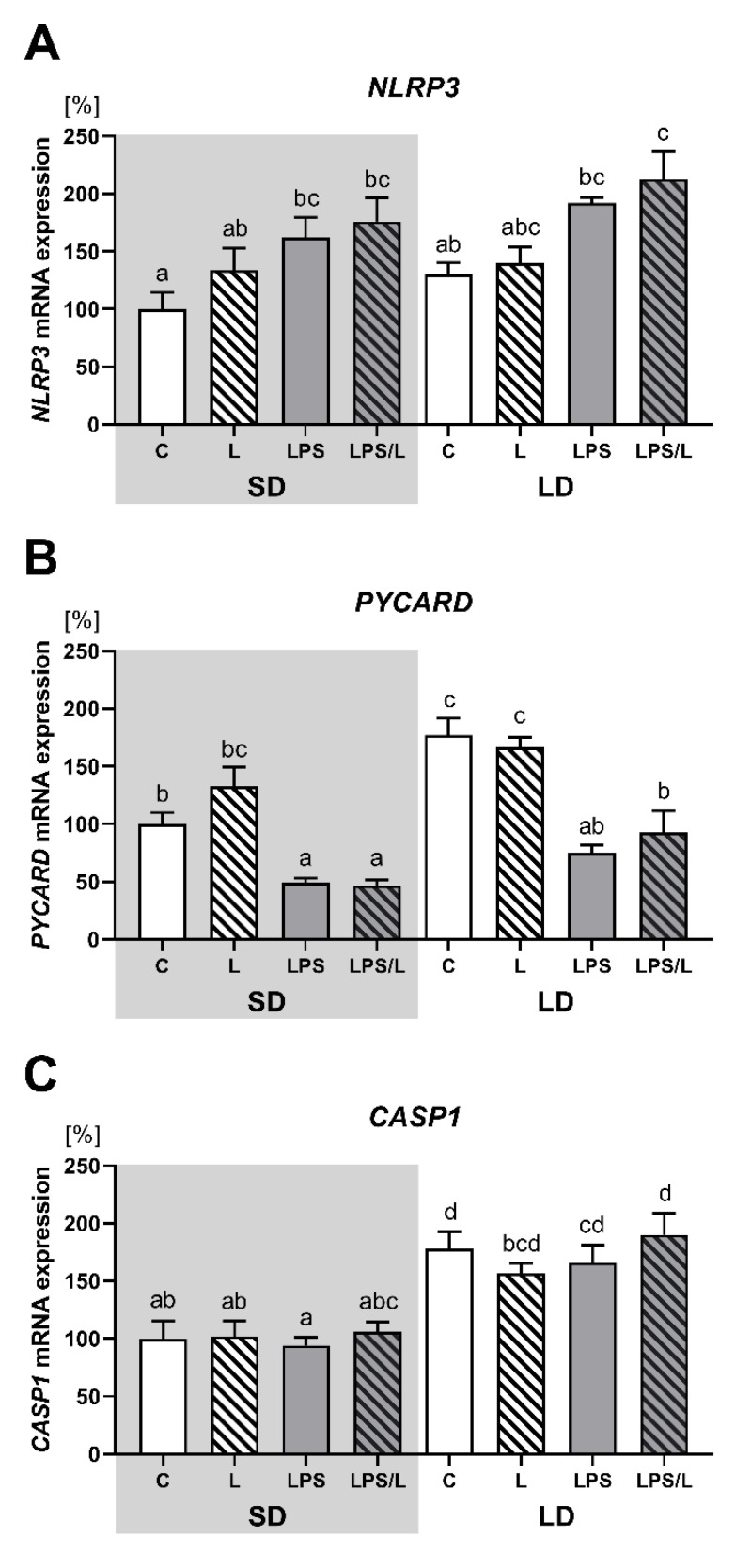
Mean (±SEM) relative *PYCARD* (**A**), *NLRP3* (**B**) and *CASP1* (**C**) mRNA expression in the ovine choroid plexus of saline (C, control—white bars), saline–leptin (L, white hatched bars), LPS (grey bars) and LPS–leptin (LPS/L, grey hatched bars) treated ewes, during the short-day (SD, dark background) and the long-day (LD, light background) photoperiod. Different lowercase letters indicate significant differences between all groups at *p* < 0.05 (two-way ANOVA—Tukey’s post hoc test). The results are presented as percentage values (%), where the SD C group is considered as 100%.

As indicated in Figure 4, there was no effect of leptin alone on NLRP3, PYCARD and CASP1 expression. The expression of NLRP3 was significantly (*p* < 0.05) higher in LPS and LPS/L groups than in the saline-treated groups (C and L group) but only during SD (Figure 4A). In turn, the expression of PYCARD was significantly (*p* < 0.05) lower in the LPS and LPS/L groups than in the saline-treated groups (C and L group) during both photoperiods (Figure 4B). There was no effect of LPS and LPS in the co-treatment with leptin on CASP1 expression (Figure 4C). Moreover, in the saline-treated groups, the expression of PYCARD and CASP1 was significantly (*p* < 0.05) higher during the LD than in SD (Figure 4C).

### 2.6. The Effect of Leptin on IL6, IL6R and IL6ST Gene Expression

**Figure 5:**
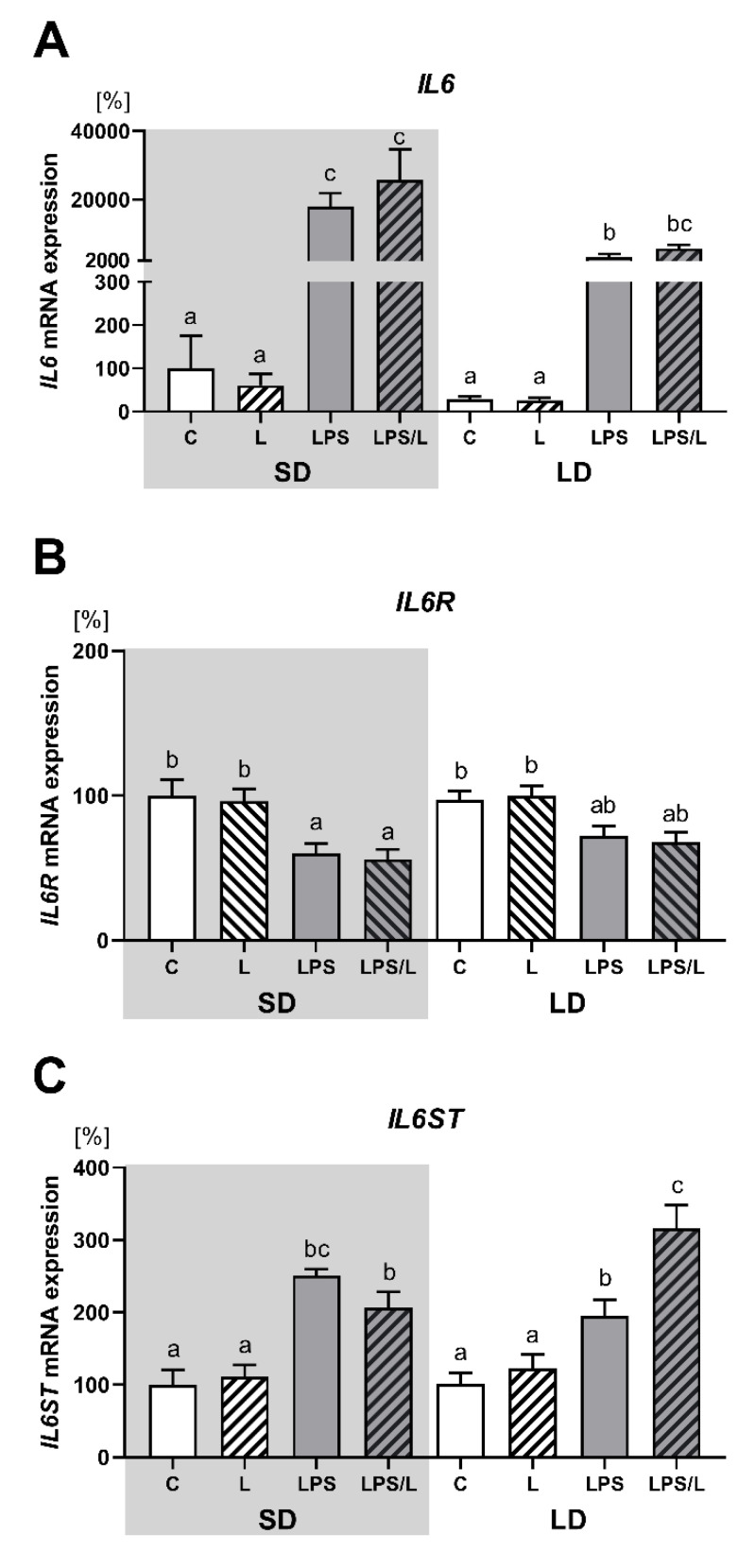
Mean (±SEM) relative *IL6* (**A**), *IL6R* (**B**) and *IL6ST* (**C**) mRNA expression in the ovine choroid plexus of saline (C, control—white bars), saline–leptin (L, white hatched bars), LPS (grey bars) and LPS–leptin (LPS/L, grey hatched bars) treated ewes, during the short-day (SD, dark background) and the long-day (LD, light background) photoperiod. Different lowercase letters indicate significant differences between all groups at *p* < 0.05 (two-way ANOVA—Tukey’s post hoc test). The results are presented as percentage values (%), where the SD C group is considered as 100%.

As indicated in Figure 5, there was no effect of leptin on the expression of IL6 and its receptors (IL6R and IL6ST) under physiological conditions. The expression of IL6 and IL6ST was significantly (*p* < 0.05) upregulated by LPS and LPS in co-treatment with leptin in both photoperiods (Figure 5A,C, respectively) and the effect of LPS on IL6 expression was significantly (*p* < 0.05) higher during SD than LD (Figure 5A). In turn, the expression of IL6R was significantly (*p* < 0.05) lower in LPS and LPS/L groups than in the saline-treated groups (C and L groups) but only during SD (Figure 5B). Moreover, the synergistic effect of leptin and LPS was revealed in the IL6ST expression but only during LD (Figure 5C).

### 2.7. The Effect of Leptin on CCL2 Gene Expression

**Figure 6:**
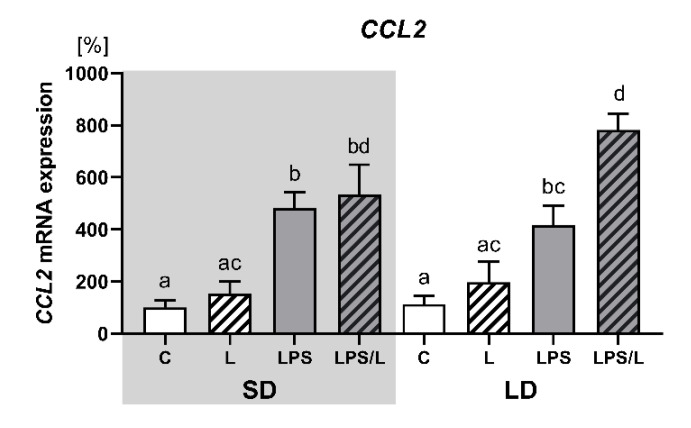
Mean (±SEM) relative *CCL2* mRNA expression in the ovine choroid plexus of saline (C, control—white bars), saline–leptin (L, white hatched bars), LPS (grey bars) and LPS–leptin (LPS/L, grey hatched bars) treated ewes, during the short-day (SD, dark background) and the long-day (LD, light background) photoperiod. Different lowercase letters indicate significant differences between all groups at *p* < 0.05 (two-way ANOVA—Tukey’s post hoc test). The results are presented as percentage values (%), where the SD C group is considered as 100%.

As indicated in Figure 6, the expression of CCL2 was not influenced by leptin alone but was significantly (*p* < 0.05) upregulated by LPS and LPS in co-treatment with leptin in both photoperiods. However, the synergistic effect of leptin and LPS was revealed only during LD.

## 3. Discussion

In the present paper, we provided the first evidence that the administration of exogenous leptin, with a raised plasma concentration to the level similar to that observed in obese sheep [44], influenced the expression of genes related to the innate immunity in the ovine ChP in a photoperiod- and LPS-dependent manner. Leptin affected the mRNA expression of examined genes in the LPS-treated but not in the saline-treated sheep. In the physiological state, the effect of leptin seemed to be tissue-specific, since leptin significantly upregulated the expression of *IL6* and *IL1R2* in the aorta [45], *IL1B*, and *IL1R2* in the perivascular adipose tissue (PVAT) [46] and *IL1B*, *IL6*, *IL6R* and *IL6ST* in the anterior pituitary (AP) [47] collected from the same sheep. In the LPS-challenged conditions, we observed the synergistic effect of LPS and leptin on the expression of *IL1B* but only during SD, and *IL1R2*, *IL1RN*, *IL6ST*, *CCL2* and *SOCS3* only during LD. Leptin also attenuated LPS-induced *IL1R1* mRNA expression but only during SD. These together indicated that the photoperiod apparently influences the leptin action on the ChP response to the LPS-induced acute inflammation. Interestingly, the lack of synergistic action of leptin and LPS on *IL6* expression observed in the ChP was also demonstrated in PVAT [46], in which leptin did not induce *IL6* mRNA expression also in a physiological state. In turn, in the aorta and AP leptin increased *IL6* mRNA expression in both physiological and LPS-challenged conditions [45,47]. One might consider the possibility that a very strong LPS-induction of *IL6* expression, observed in the ovine ChP, masked the potential impact of leptin. It should be mentioned, that leptin had no effect on *TLR4*, which is known as an LPS receptor [48]. However, *TLR4* expression increased under LPS-challenge, what was in agreement with our previous studies [49]. This increase was stronger during SD than LD, which was discussed in our recent work [21].

It is generally accepted that leptin signalling depends on the presence of LEPRb, which signals mainly through the JAK2/STAT3 pathway inducing SOCS3, which inhibits leptin signalling via a feedback mechanism. We used *SOCS3* mRNA expression as a marker of the leptin stimulation of the JAK2/STAT3 pathway. In the ovine ChP, leptin receptors are expressed at a fairly high level, since the Cq (cycle quantification) value ranged from 22.4 to 25.6 for *LEPRa* and from 21.7 to 25.8 for *LEPRb* (Supplementary Figure S3). *LEPRa* was expressed in all investigated groups on a similar level with the exception of control groups in which the expression of *LEPRa* was higher in LD than SD photoperiod, which might explain higher amounts of leptin entering CSF after intravenous leptin injection during LD [25]. According to Bernabucci et al. [50], the increase in the expression of *LEPRa* and *LEPRb* in adipocytes in cows in response to the LD photoperiod may be related to the influence of prolactin. A similar mechanism may be suggested in sheep since prolactin receptors are abundant in the ChP [51] and prolactin concentration in the blood plasma was higher under LD in the present study. *LEPRb* was also expressed in all groups, but its expression was not modified by photoperiod but it was attenuated by an LPS-challenged. This was in contrast to the significant effect of leptin observed in LPS-treated animals. This may be due to the fact that leptin was administered 30 min after LPS treatment and therefore acted on an unchanged number of the LEPRb protein. In the physiological state, despite two times higher plasma leptin concentration during LD than SD in the control (1.2 ± 0.1 vs. 0.6 ± 0.1 ng/mL) sheep, we did not observe differences in *SOCS3* expression. Leptin administration also did not increase *SOCS3* expression, which may indicate a similar level of JAK2/STAT3 activation during both photoperiods. Beside leptin, *SOCS3* expression is stimulated by numerous factors including LPS, IL-6 [52,53]. Indeed, under LPS challenge conditions, the expression of *SOCS3* in the ovine ChP paralleled the increase in the expression of *IL6* which was higher during SD than LD. The synergistic action of LPS and leptin on *SOCS3* observed only during LD may explain the lack of leptin effect on *IL1B* expression observed in the LPS/L group at the same time. In turn, the lack of *SOCS3* elevation during SD seemed to be responsible for the increase in *IL1B*. Studies by Qin et al. [52] indicated a rapid expression of SOCS-3 protein, which was detectable already 1 h after LPS stimulation in murine macrophage and microglial cell lines and primary microglia. The induction of *IL1B* expression is a priming step, as synthetised pro-IL-1β has to be cleaved by caspase-1 to become an active molecule. The activation of caspase-1 occurs via the recruitment to a multi-protein complex termed the inflammasome composed by NLRP3, the PYCARD molecule containing the caspase recruitment domain allowing for the recruitment, oligomerization and simultaneous activation of caspase-1 [54]. In the ovine ChP, we observed the expression of all inflammasome elements. As expected, the expression of *NLRP3* was upregulated by LPS, however, during LD, it was not as strong as in SD, which may be linked with the attenuated ChP response to LPS-induced acute inflammation, which was discussed in our recent article [21]. IL-1β acting through its signalling receptor IL1R1 seemed to be responsible for the upregulation of *IL1R1*, *IL1R2*—a cytokine decoy receptor and receptor antagonist—*IL1RN* in the ovine ChP in the LPS challenge since similar upregulation was observed in the endometrial epithelial cells following IL-1β treatment [55]. While the studies of Bellehumeur et al. [55] demonstrating that IL-1β in a dose of 10 ng/mL is less effective in *IL1R1*, *IL1R2* and *IL1RN* induction than 1 ng/mL, one could explain the attenuation of LPS-induced *IL1R1* mRNA expression in the ovine ChP in LPS/leptin group, as it cannot be applied to *IL1R2* and *IL1RN*, whose expression did not differ between the LPS and LPS/L group during SD. Leptin is capable of inducing the expression and secretion of IL-1RN by human monocytes [56], which may explain the upregulation of *IL1RN* in the LPS/L group observed in the ovine ChP during LD. Keeping in mind that the balance between IL-1β and IL-1RN and IL-1R2 plays an important role in the regulation of inflammatory responses [57], the selective leptin stimulation of *IL1R2* and *IL1RN*, but not *IL1B*, observed in our study may suggest an anti-inflammatory action of leptin in the ChP during LD. Moreover, when we considered the synergistic effect of leptin and LPS on *IL6ST* expression, we found that it may be related to the inducing effect of IL-10 [58], the secretion of which may be stimulated by leptin [59].

The ChP is considered as a gateway to the CNS and is uniquely structured to support and regulate immune cell trafficking. In the opinion of Meeker et al., [60], in the initial stage of inflammation, the macrophages trafficking into the CSF through the ChP play a key role in the development of the CNS immune response. This point of view is also supported by the fact that the ChP is characterised not only by the residing of the large stroma population of macrophages but also by the presence on the apical surface of ChP epithelium, the macrophages described as epiplexus or Kolmer cells [61]. The significance of the role of monocytes in CNS is evidenced by the fact that in the basic conditions in human CSF, monocytes constitute as much as 23% of the leukocyte population, while in blood it is only 6.5% [60]. The CCL2 chemokine, formerly named monocyte chemoattractant protein-1, is expressed in the ChP at the highest level among the CNS structures of rats and is highly upregulated during peripheral tissue inflammation [62]. Therefore, the discovery that LPS-induced recruitment into the mouse brain of neutrophils, the activation and migration of which usually follow the activation of monocytes/macrophages is regulated by leptin [40], prompted us to analyse the *CCL2* expression in the ChP. In fact, we again did not observe the significant leptin impact on the *CCL2* expression under the basal conditions, but we demonstrated that LPS caused expression induction, in both photoperiods, and during LD, we observed the synergistic upregulation caused by leptin and LPS treatments. The LPS induction of *CCL2* expression in the human monocytes/macrophages is mediated by TLR4/MyD88 (myeloid differentiation factor 88) pathway, which for further transfer of the signal requires the recruitment of IRAK proteins [63]. Therefore, we might suggest that the synergism of leptin and LPS action, observed in *CCL2* expression, may be related to the leptin upregulation of the IRAK1 protein observed in monocytes/macrophages [64]. It should be mentioned, that there is a correlation between *CCL2* mRNA expression in the rats ChP and the level of CCL2 protein in the CSF [65].

In summary, our study on an ewe model demonstrated the pro-inflammatory action of pleiotropic adipokine—leptin on the ChP. This effect revealed only under the LPS-induced immune/inflammatory challenge, was photoperiodically modulated and observed in sheep in which the plasma concentration of leptin was elevated to the level found in obese animals. The latter may indicate a higher induction of the ChP response to LPS-treatment in obese rather than normal subjects. The strong synergistic action of leptin and LPS on *IL1B* synthesis, at least at the mRNA level, may be of particular interest for the study of mechanisms responsible for the central inhibition of reproductive processes by immune stress induced by infections and inflammatory diseases in animals species. In rats and sheep, central IL-1β was demonstrated to be an important negative regulator of gonadotropin releasing hormone biosynthesis in the hypothalamus [66,67], as well as *IL1B* and *IL1R1* expression which was upregulated by the LPS treatment [68]. In our previous study, we demonstrated that at the beginning of LPS-induced inflammatory challenge, the local synthesis of IL-1β in the ovine ChP but not its transport throughout the blood–CSF barrier is an important source of IL-1β in the CSF from where it can propagate its synthesis in the brain parenchyma [69]. Further studies are necessary to confirm these results on the protein level.

## 4. Materials and Methods

### 4.1. Animals and Experimental Design

The animal experiments were conducted with the approval of the Third Local Ethical Commission of Warsaw University of Life Sciences—SGGW (Warsaw, Poland, authorisation number 56/2013) and were carried out on the frozen ChP samples collected from adult female blackface sheep (*n* = 48, 2 years old) consistent with the studies of Krawczyńska et al. [46]. The experiments were performed during two photoperiods: in December (SD season; day: night 8:16) and in June (LD season; day: night 16:8). The ewes were kept indoors under natural lighting conditions (latitude 52° N, 21° E) and were fed a consistent diet of commercial concentrates with hay and water available ad libitum, according to the recommendations of the National Research Institute of Animal Production. During the experiments, the ewes were kept in individual pens and to reduce social isolation, visual contact with other members of the flock was maintained. During the reproduction season in SD, the oestrous cycle was synchronised by the Chronogest^®^ CR (Merck Animal Health, Boxmeer, The Netherlands) method previously described in detail by Krawczyńska et al. [46]. During the LD period, the ewes were in seasonal anoestrous, and therefore they did not require synchronization. In the experiments conducted during SD and LD, the ewes were randomly divided into four groups (*n* = 6 in each): treated intravenously with saline (control group, C), saline plus leptin (L, the recombinant sheep leptin (Protein Laboratories Rehovot (PLR) Ltd., Rehovot, Israel)) in a dose of 20 μg/kg of body mass, LPS (LPS from Escherichia coli 055:B5 (Sigma-Aldrich, St. Louis, MO, USA)) in a dose of 400 ng/kg of body mass and LPS plus leptin (LPS/L). The leptin solution was injected 30 min after LPS treatment. Three hours after LPS/saline, which is equivalent to 2.5 h after leptin treatment, the ewes were sacrificed. After decapitation, the brain was removed and the ChP from lateral ventricles was collected, snap-frozen in liquid nitrogen, and stored at −80°C. During both experiments, the body temperature was measured before and every 30 min after LPS administration and blood samples were collected every 30 min through the jugular vein catheter, starting 1 h before LPS/saline treatment and continuing until the animals’ scarification, for cortisol, prolactin and leptin measurement.

### 4.2. Hormone Concentration Measurement

Plasma cortisol and prolactin concentrations were determined according to the method previously described for cortisol [49] and prolactin [70]. The sensitivity of the assay for cortisol and prolactin was 0.95 and 2 ng/mL, and the intra- and inter-assay coefficients of variation were 10 and 12%, and 9 and 12%, respectively.

Plasma leptin concentration was determined by double-antibody, ovine-specific radioimmunoassay (RIA) developed by Delavaud et al. [71], where the sensitivity of the assay was 0.3 ng/mL, and the intra- and inter-assay coefficient of variation were 2.4 and 10.7%, respectively.

### 4.3. Relative mRNA Expression

Frozen ChP samples were homogenised with using FastPrep24 instrument (MP Biomedicals, Illkirch-Graffenstaden, France) and dedicated to Lysing Matrix D tubes (MP Biomedicals, Illkirch-Graffenstaden, France), filled with lysing buffer (RA1) from the NucleoSpin RNAII Kit (MARCHEREY-NAGEL, Düren, Germany) for total RNA isolation. According to the manufacturer’s protocol, during RNA isolation, the genomic DNA digestion step was carried out. The concentration and purity of the obtained RNA were evaluated spectrophotometrically by using a NanoDrop 1000 instrument (Thermo Fisher Scientific, Waltham, MA, USA). RNA integrity was controlled by electrophoresis method with using 1.2 % agarose gel containing the GelRed Nucleic Acid Gel Stain (Biotium, Fremont, CA USA). The reverse transcription (RT) of total RNA was performed using DyNAmo cDNA Synthesis Kit (Thermo Fisher Scientific, Waltham, MA, USA), according to the manufacturer protocol and each RT reaction contained 1µg of total RNA. Obtained cDNA was frozen and kept at −20 °C until further analysis.

For the real-time PCR analysis, the specific primers pairs were selected based on the literature data or were originally designed using Primer-BLAST (National Center for Biotechnology Information, Bethesda, MD, USA). All primers were synthesised by Genomed (Warsaw, Poland) and their sequences and source data are listed in Table 1.

The real-time PCR analysis was performed using the Viia7 instrument (Applied Biosystems by Life Technologies, Waltham, MA, USA) and each real-time PCR reaction contained 3 µL cDNA (1:10), 0.2 µM of each oligonucleotide from a specific pair and 5 µL of DyNAmo SYBR Green qPCR kit with ROX (Thermo Fisher Scientific, Waltham, MA, USA). The cDNA samples and reaction mix were transferred into 384-well plates, by the Bravo Automated Liquid Handling Platform (Agilent Technologies, Santa Clara, CA, USA). The following protocol was used: 95 °C for 10 min for the hot start modified Tbr DNA polymerase, and 40 cycles at 95 °C for 15 s (denaturation), 60 °C or 55 °C for 30 s (primer annealing), and 72 °C for 30 s (extension). After the cycles, a final melting curve analysis was performed under continuous fluorescence measurement to assess the specificity of the amplification. The primer annealing temperature was evaluated empirically by gradient PCR analysis (Labcycler SensoQuest, Göttingen, Germany) and for almost all primer pairs was set at 60 °C and only for TLR4 at 55 °C.

Three housekeeping genes were examined: glyceraldehyde-3-phosphate dehydrogenase (GAPDH), β-actin (ACTB) and histone deacetylase 1 (HDAC1) using free NormFinder software (MOWA, Aarhus, Denmark). For the presented experimental conditions, we identified HDAC1 as the best reference gene, and HDAC1 and ACTB as the best combination of two genes, which was used in further analyses. Expression analysis was performed with using the Real-Time PCR Miner (available online: http://ewindup.info/miner/), a software based on the Zhao and Fernald [78] algorithm, that allows considering the actual reaction efficiency for each pair of primers for every single reaction.

### 4.4. Statistical Analysis

Statistical analyses were performed on raw data after the verification of normality assumptions (Shapiro–Wilk test). Before the analysis, the data that failed the normality test were subjected to logarithmic transformation. To determine the differences in hormone levels before and after treatments, cortisol, prolactin and leptin concentrations values were subjected to repeated measures analysis of variance (ANOVA) followed by Sidak’s multiple comparisons test. The differences in the hormones concentration between photoperiods were calculated separately for the samples collected before and after treatments. These data and results of real-time PCR were analysed by applying two-way ANOVA followed by post hoc Tukey’s test and presented as the mean ± standard error of the mean (SEM) with statistical significance considered at *p* < 0.05. The results of real-time PCR analysis were presented as the mean of relative gene expression (±SEM) vs. the geometric mean of HDAC1 and ACTB expression, validated as the best reference genes combination. The real-time PCR results shown in the figures were converted into percentages, where the values for the SD saline/saline-treated group were considered as 100%. All statistical analyses were performed in the Graph Pad PRISM 8 (Graph Pad Software, San Diego, CA, USA).

## Figures and Tables

**Table 1:** Sequences of oligonucleotide primers used for real-time PCR.

Gene	(Forward/Reverse) Sequence 5′→3′		Amplicon Size (bp)	References/Sources
*LEPRa*	F: TGCTGTCACCCAGTGATTACAATC	R: CAAAGTATGTCCGTTCTCTTCTGA	412	[72]
*LEPRb*	F: AACTACAGATGCCCTGCTTTTGAC	R: CCTTTGGTGGAGAATGGTTGC	257	[72]
*SOCS3*	F: TTTCTCGTAGGAGTCCAGGTG	R: CCCCCAGGAGAGCCTATTAC	140	XM_027974200.1
*TLR4*	F: TGGATTTATCCAGATGCGAAA	R: GGCCACCAGCTTCTGTAAAC	152	[73]
*IL1B*	F: CAGCCGTGCAGTCAGTAAAA	R: GAAGCTCATGCAGAACACCA	137	[74]
*IL1R1*	F: GGGAAGGGTCCACCTGTAAC	R: ACAATGCTTTCCCCAACGTA	124	[74]
*IL1R2*	F: CGCCAGGCATACTCAGAAA	R: GAGAACGTGGCAGCTTCTTT	162	[74]
*IL1RN*	F: AGGATCTGGGATGTCAACCA	R: CATGGATCCCCAGGAACATA	145	[74]
*NLRP3*	F: CCGTCTGGGTGAGAGCGTGAA	R: TCCTGTTGGCTCCTGTGTTCCT	78	[75]
*PYCARD*	F: GCCGTGGACCTTACCGACAA	R: GCAGTCCTGGCTTGGCTATCTT	110	[75]
*CASP1*	F: GGATACAATAAATGGCTTGCTGG	R: CTCGGGCTTTATCCATAGTTGT	196	[75]
*IL6*	F: GTTCAATCAGGCGATTTGCT	R: CCTGCGATCTTTTCCTTCAG	165	[74]
*IL6R*	F: TCAGCGACTCCGGAAACTAT	R: CCGAGGACTCCACTCACAAT	149	[74]
*IL6ST*	F: GGCTTGCCTCCTGAAAAACC	R: ACTTCTCTGTTGCCCACTCAG	139	[76]
*CCL2*	F: CTCGCTCAGCCAGATGCAAT	R: AGGTTGGGGTCTGCACAAAA	173	XM_004012471.3
GAPDH *	F: TGACCCCTTCATTGACCTTC	R: GATCTCGCTCCTGGAAGATG	143	[77]
ACTB *	F: GCCAACCGTGAGAAGATGAC	R: TCCATCACGATGCCAGTG	122	[77]
HDAC1 *	F: CTGGGGACCTACGGGATATT	R: GACATGACCGGCTTGAAAAT	115	[77]

*LEPRa—*leptin receptor short isoform; *LEPRb—*leptin receptor long isoform; *LEPR—*leptin receptor all isoforms; *SOCS3—*suppressor of cytokine signalling 3 (originally designed/1 coding exon); *TLR4—*Toll-like receptor 4; *IL1B—*interleukin 1-beta; *IL1R1—*interleukin 1 receptor, type I; *IL1R2—*interleukin 1 receptor, type II; *IL1RN—*interleukin 1 receptor antagonist; *NLRP3—*NLR family pyrin domain containing 3; *PYCARD—*PYD (pyrin domain) and CARD (caspase activation and recruitment domain) domain-containing; *CASP1—*caspase 1; IL6—interleukin 6; *IL6R—*interleukin 6 receptor; *IL6ST—*glycoprotein 130; *CCL2—*C-C motif chemokine ligand 2 (originally designed/F:149/150 exon span); * reference genes: *GAPDH—*glyceraldehyde-3-phosphate dehydrogenase; *ACTB—*beta-actin; *HDAC1—*histone deacetylase1.

## Supplementary Materials

Supplementary Materials can be found at https://www.mdpi.com/1422-0067/21/20/7647/s1.
