# Supplementary material for: Photoperiod Affects Leptin Action on the Choroid Plexus in Ewes Challenged with Lipopolysaccharide—Study on the mRNA Level
Source: Int J Mol Sci. 2020 Oct 15;21(20):7647. doi: 10.3390/ijms21207647 (PMC7589540; doi:10.3390/ijms21207647)
Supplement: Supplementary file 1 [file ijms-21-07647-s001.pdf]

Supplementary Materials

Supplementary Figure S1: Body temperature values throughout the experiments.

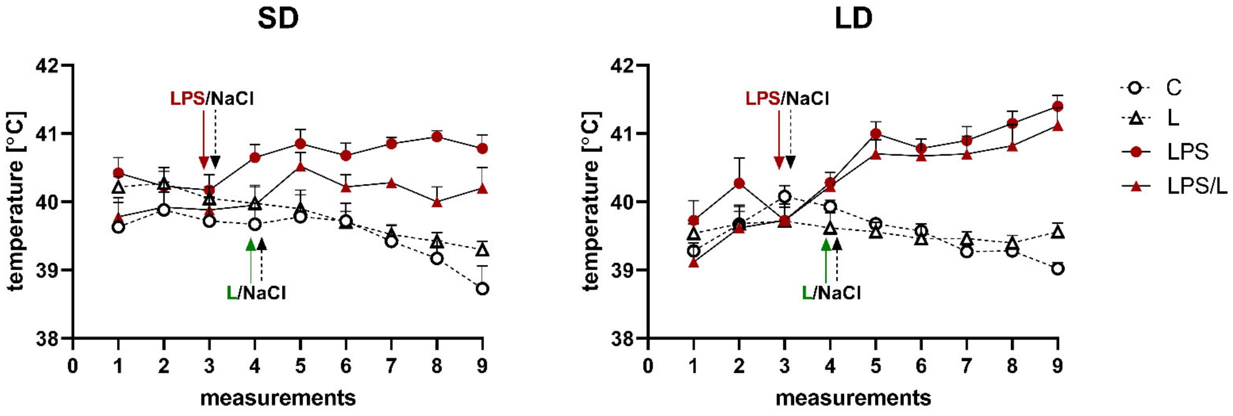

Figure S1. Mean (±SEM) body temperature values measured in ewes every half hour throughout the experiments carried out during short-day (SD, left panel) and long-day (LD, right panel) photoperiod.

Supplementary Figure S2: mRNA expression of *TLR4*

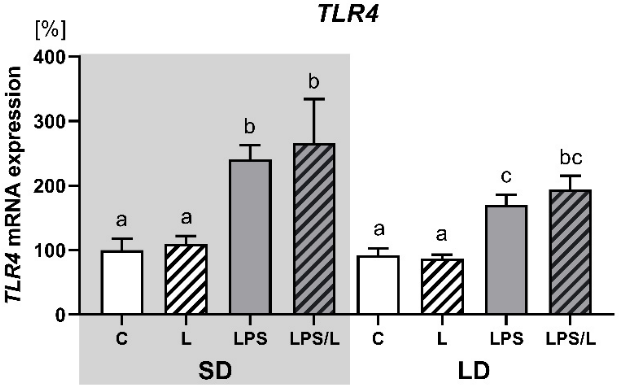

Figure S2. Mean (±SEM) relative TLR4 mRNA expression in the ovine choroid plexus of saline (C, control – white bars), saline-leptin (L, white hatched bars), LPS (grey bars) and LPS-leptin (LPS/L, grey hatched bars) treated ewes, during short-day (SD, dark background) and long-day (LD, light background) photoperiod. The results are presented as percentage values [%], where SD C group is considered as 100%.

Supplementary Figure S3: Cycle quantification values of LEPRa and LEPRb expression.

Cq (cycle quantification) values obtained from raw real time PCR data analyzed with using Real-Time PCR Miner software:
LEPRa – Cq range from 22.4 to 25.6; mean (± SEM) 23.2 ± 0.1
LEPRb – Cq range from 21.7 to 25.8; mean (± SEM) 23.7 ± 0.1
